# 

**Published:** 1862-08

**Authors:** 


Affections of the Eye, - - - 4, 294

Affections of the Uterus, - - - 5, 291

Amputations in the Field, - 23

Abstract of the Proceedings of the Buffalo Medical Association, 11, 41, 74, 97, 129 161, 193, 238, 268, 299, 338, 369.

Abstract of the Proceedings of the Racine Medical Association, 201, 243, 264, 335

Analysis of 100 Ophthalmic Cases, - 211

Address by Prof. Thomas F. Rochester, 225

Amputations, - - - - - 246

Abscess, - - - - - - 259

Action of Opium, - - - - - 306

Appointments, - - - - - 32, 64

Ascetes, case of - - - - 200

Address, extracts from. By Prof. Jas. P. White, - - - - - 135

American Journal of Ophthalmology, 30, 283

Appointment of Delegates to American Association, - - - 308

Annual Report of Surgeon General, - 185

American Medical Monthly, - - 290

American Wine, - - - - - 322

Abbott's History of the Civil War, - 353

Alcohol, Action and Uses of - - 356

Aneurism, - - - - - - - 369

Bibliographical Notices.—Walshe on Diseases of the Heart and Great Blood Vessels, - - - - 56

Caries of Elbow-joint. By N. C. Husted, - - - - - - 60

Military Surgery. By L. Bandens. 28

Advice to Mothers. By Pye Henry Chuoasse, - - - - - 29

Ranking's half-yearly Abstract, - 97

Variola, its Nature and Treatment, - 120

Books on the War, - - - - 124

Health, its Friends and Foes. By R. D. Mussey, - - - - - 154

Clinical Treatise on Diseases of the Liver. [Sydenham Publications,] 156

New Book of Medicine and Surgery, - 156

Hospital Steward's Manual, - 157

Anatomy of the Arteries. - - 157

Medical Provision for Railroads. - 179

Headland on the Action of Medicines, 183

Consumption in New England, - 221

Braithwaite's Retrospect, - - 222

Gunning S. Bedford on Obstetrics, - 253

Diseases of Women and Children. By Gunning S. Bedford, - - 316

Wilson on Diseases of the Skin, - 317

Cazeaux's Midwifery, - - 318

Meig's Treatise on Obstetrics, - - 350

Paih's Institutes of Medicine, - 352

Packard's Minor Surgery, - - 320

Medical Student's Vade Mecum, - 320

Brand & Taylor's Chemistry, - -

Bowman's Medical Chemistry, - 320

Report on Medico-Legal Contributions, 27

Benign Tumor, - - - - - 36

Brigade Surgeons in the Army, - - 30

Braithwaite's Retrospect, - - 222

Buffalo City Dispensary for 1863, - 191

Congress, Dr. Morton asking pay for ether, - - - - - 224

Congress, Homœpathists in, - - 232

Commencement of Lectures, - 128

Cystitis in a young man, - - - - 6

Caries of the Knee-joint, - - 37

Case of Monstrosity, - - - 97

Conservative Medicine, - 101, 269

Case of Neuroma, - - - - 172

Case of Ascites, - - - 200

Commencement Exercises, - - 249

Catalogue of Books, - - - 290

Canada Lancet, - - - - 322

Calomel and Tartar Emetic stricken from supply table, - - - - 354

Concentrated Medicines, - - - 289

Calculus Urinary, - - - - 371

Diphtheria, treatment of, - - 212

Diseases of the Army. By S. B. Hunt, M. D. 202

Diphtheria, Notes upon, - - - 204

" in Boston, Erie Co. - - 122

" in Collins and Concord, 154

Diseases of Bones and Joints, - - 263

Detection and use of Alcohol, - 303

Death of Sir Benjamin Brodie, - - 158

" Prof. Cooper, - - 160

" Thomas Wakely, - - 32

" Richard Charles, - - 321

" A. S. Sprague, - - 215

" Charles H. Wilcox, - - 143

" Lieut. James C. Mackay, - 127

" Josiah Trowbridge, - 94

" Charles Heartman, - - 354

Davis' Splint for Morbus Coxarius, - 152

Effects of the Preperation of Iron, - 385

Editorial, - - - - 27, 53, 89, 118. 249, 279, 304, 344, 143, 179, 212, 377

Erysipelas, - - - - - 68, 296

Examining Recruits in Buffalo, - - 364

Erie County Medical Society, - 348

Exsection of a portion of the malar bone, 173

Electro Therapeutics. - - - - 296

Editors of Medical Journals and the so-called Dermatobiotikon. - - 290

Employment of Telegraph Sutures, - 288

Fractures, - - - - - - 53, 35, 261

Fungi of Wheat Straw, - - - - 83

Gonorrhœa, - - - - - 7, 293

Glass in foot 15 years, - - - 80

Gun-shot Wounds, - - - - 260

Hand Litters for the Army, - - 183
Hernia, Strangulated Intestine removed 220
" Inguinal, operation for, - 8
" Strangulated, operation for, radical cure. - - - 341

Institute for treatment of Deformities, 123
Inguinal Hernia, - - - - 8
Inflammation of the Knee-joint, - - 38
Inspection of Hospitals, - - 152
Injections in treatment of Uterine Disease, 286

Knee-joint, inflammation of, - - 38

Letter from Dr. Strong, - - 147

Lint and Oakum in Gun-shot wounds, 118

Letter from Camp Belger, - - 119

Lacerations, - - - - 295

Lithotomy, - - - - - 373

Medical and Surgical History of the Rebellion, - - - - 32
Military Surgery, - - - - 53
Malignant Tumor, - - - 71
Medical Provision for Railroads, - 179
Monstrosity, case of, - - - 97
Meeting of State Medical Society, - 254
Meeting of American Medical Association, 258, 279, 344, 381.
Medical Regulations during Battle, - 311
Materia Medica and Hygiene in University of Buffalo, - - - 354

Notes on Army Medical Service. By S. B. Hunt, M. D. - - - 22
Necrosis, - - - - - - 39
Notes of Surgical Cases. By J. F. Miner, M. D. - - - - 45
New Remedies. By Sam'l R. Percy, M. D. 50
Notes of Surgical Cases. By S. Barrett, M. D. - - - - - 170
Neuroma, case of, - - - - 172
New York Ophthalmic School, - 282

Observations connected with Measles, 80
Ophthalmic Cases, - - - 211
Obituary, Death of Lieut. James Mackey, 127
" Death of Dr. R. Charles, - 321
" Death of Dr. C. Heartman, - 354
Ossium, Grygilitas - - - 376

Presedtation of the Umbilical Cord, 11
Paralysis, - - - - - 65, 293
Poisoning by Opium, - - - 79
Pelvic Hæmatocele, - - - 108
Presentation to Dr. Hunt, - - 64
Promotion of Buffalo Surgeons, - 123
Perforation of Intestines by worms, 168
Providence Insane Asylum, - - 150
Publications of Medical Books, - 309
Permanent Rooms for Buffalo Medical Association, - - - 315
Prospects of the American Medical Association, - - - - 321
Polypi-Uterine, - - - - 129
Physicians' Hand Book of Practice, - 126
" Visiting List, - - 126
" Pocket Memorandum, - 127
Peritonitis Acute, - - - - 375
Proceedings of the Genesee County Medical Society, - - - - 373

Report of Surgical Cases at Buffalo Gen'l Hospital. By J. R. Lothrop, M. D. 3, 33, 65
Retro Uterine Hæmatocele. By James P. White, M. D. - - - - 12
Removal of left half of Inf. Maxillary Bone, by S. Barrett, M. D., - - - 170
Resection of Elbow, - - - 171
Report of Deaths in the City of Buffalo. By S. Eastman, M. D. 32, 64, 128, 160, 192
Report on Exemption in Erie Co. By Drs. J. S. Trowbridge and J. E. King, Ex. Surgeons, - - - - - 176
Report on Exemption in Oneida Co. By Dr. Ira D. Hopkins, Ex. Surgeon, 177
Rupture of Urethra. By S. Barrett, M. D. 199
Report of Surgical Cases in Buffalo Gen'l Hospital. By J. F. Miner, M. D. 246, 259, 291.
Report of Medical Cases at the Sisters of Charity Hospital. By J. R. Lothrop, M. D. - - - - - 324
Resignation of Prof. H. H. Childs, - 315
Racine Medical Association, - 192

Spermatorrhœa, - - - [illegible]7
Spasms from Intestinal Irritation and Teething, - - - - 16
Synovitis, - - - - - 38
Strabismus, - - - - - 46
Syphilis, - - - - 69, 293
Strangulated Hernia, operation for. By J. F. Miner, M. D, - - 341
Selections—Extirpation of diseased eye to preserve healthy one, - - 63
Snbcutaneous injection of Quinine, 217
Typh. Fevers, - - - - 217
Typhoid and Scarlet Fever, - 218
Chlorine and Chlorine Acids, - 218
Zymotic Diseases, - - - 219
Delirium Tremens, - - - 219
Croup, Veratrum Viride, - - 220
Pleurisy, - - - - 220
Surgeon C. H. Wilcox on sick leave, 124
Sanitary Commission, - - - 310
Statistics of the Globe, - - 284
Surgeons for the New Levy, - - 61
Strangulated Hernia, Intestine, removed. By H. B. Moore, M. D. - - 220
Strangulated Hernia, removal of sac, 341

Treatment of Tetanus, by J. F. Norton, M. D., U. S. N., - - - 19
Treatment of Diphtheria, - - 212
Tumor in lumbar region - - 142

Uterine Polypi, - - - - 129
Ulcers, Varicose, - - - 36
Urinary Calculus, - - - - 374

Varicose Ulcers, - - - - 36
Varicose Veins, - - - - 264
Volunteer Surgeons, - - - 64
Variola, its dature and treatment, - 121

Wounds of the Scalp, - - - 4

### BOOKS AND PAMPHLETS RECEIVED.

*A Practical Treatise on the Diseases of the Heart and Great Vessels, including the Principles of Physical Diagnosis, by* WALTER HAYLE WALSH, M. D., *Fellow of the Royal College of Physicians; Professor of the Principles and Practice of Medicine and of Clinical Medicine in University College, London; Consulting Physician to the Hospital for Consumption. A new American, from the third revised and much enlarged London edition. Philadelphia:* BLANCHARD & LEA, 1862.

*Caries of Elbow Joint, Operation of Excision with recovery of useful arm; presented to Medical Society of State of New York, at its annual meeting*, 1862, *by* N. C. HUSTED, M. D., *New York City. Albany:* C. VAN BENTHUYSEN, 1862.

*Testimony in the matter of the application of* B. FRANK PALMER, *for the extension of his patent for an Artificial Leg. Read before the the Hon. Commissioner of Patents. October* 22, 1860; *extension granted Nov.* 3. 1860. *Philadelphia:* C, SHERMAN & SON, 1862.

*Memoir of Hon. John Miller, M. D., late of Truxton, Courtland County, N. Y., by* GEORGE W. BRADFORD, M. D., *Secretary of Courtland County Medical Society. Albany*: C. VAN BENTHUYSEN, 1862.

